# Applying AVWEWM to ethical decision-making during autonomous vehicle crashes

**DOI:** 10.1038/s41598-024-54363-8

**Published:** 2024-02-16

**Authors:** Guoman Liu, Yufeng Luo, Jing Sheng

**Affiliations:** 1 Key Innovation Center of Industry and Higher Education Integration of Jiangxi Province, East China Jiaotong University, Nanchang, 330013 China; 2 School of Mechanical Engineering, Nanchang Institute of Technology, Nanchang, 330099 China

**Keywords:** Autonomous vehicle, Decision-making, Ethical dilemma, EWM, Electrical and electronic engineering, Mechanical engineering

## Abstract

At present, a few scholars studied influencing factors, rules and mechanisms of decision-making in ethical dilemmas. Many factors have been identified, and a few rules and mechanisms have been proposed. However, due to the inability to evaluate the weight and role of each factor in decision-making, it is difficult to establish a computational decision-making model to solve ethical dilemmas. Therefore, entropy weighted method (EWM) and Attribute Value Weighted EWM (AVWEWM) are used to process 84 dilemmas respectively to evaluate the weight and role of each factor in decision-making, then decision-making models based on EWM and AVWEWM are constructed to make decisions during autonomous vehicle (AV) crashes respectively. Lastly, 40 dilemmas are designed to test both decision-making models. The test results indicate that both can make clear decision-making in 40 dilemmas. However, the decision-making by AVWEWM is more consistent with public opinion than EWM. In addition, according to the weight and role of each factor in decision-making, it can also provide a few references for traffic management and legal departments to formulate traffic laws and regulations for AV in the future.

## Introductions

Autonomous driving technology is an important development trend in the future, which may subvert traditional travel mode^1^. Furthermore, it can not only facilitate driving, but also reduce the occurrence of traffic accident, improve the efficiency of traffic operation, and reduce energy consumption by sensitive perception and effective control^2,3^. Therefore, many institutions and car manufactures are engaged in development and research of autonomous driving technology. For example, Google set up WAYMO corporation to carry out road testing for many years. Tesla, NIO and other manufactures have commercialized L2 or L3 level AV to achieve good application. However, self-driving technology can reduce traffic accident, but it can not fully avoid accident because of hardware or software failure. So AV must meet moral dilemma in real life. Although it appears with low probability, AV needs to make right decision-making in a moral dilemma. Or else, people may distrust in AV, and hinder application and development of self-driving technology. Therefore, it is necessary to solve ethical dilemma for AV.

In the current time, many institutes and scholars have studied and explored influencing factors, rules and mechanisms of decision-making in ethical dilemmas. For example, Massachusetts Institute of Technology (MIT) deployed Moral Machine, an online open experimental platform, designed to explore how human make decision when AV faces ethical dilemmas. The survey results, published in Journal of Nature in October 2018, show three preferences: to protect human, to protect more lives, and to protect younger^4^. However, Kochupillai et al. believed that applying human preferences to decision-making in ethical dilemmas, brings some discrimination, which lead to the infringement of rights and interests of some people and violate fairness^5^. Furthermore, Hubert Etienne also believed that applying these survey results in Moral Machine to decision-making in ethical dilemma, can improve human’s understanding of decision-making, but cannot impose human preferences on AV^6^. Professor Su thought that decision-making in ethical dilemma should be considered from laws and regulations, technical supervision, ethical modeling and procedural justice for AV^7^, because laws and regulations are used to restrict and supervise decision-making, and designer and developers’ morality should be taken into account in decision-making. Furthermore, some experts thought that AV should make decision in ethical dilemma by a moral rule, such as utilitarian, deontology. Good or bad of decision-making is measured by number in utilitarian, which believes that “the greatest happiness is the greatest number”^8^. It ensures that decision-making can achieve maximum utility and minimum harm in ethical dilemmas, which is easy to be accepted by people. However, decision-making by utilitarian sometimes violates the rights and interests of some pedestrians who obey rules and have safe facilities, it is unscientific and unreasonable. So Kant’s deontology requires that decision-making should be made by moral principle or legitimacy rather than utility and consequence^9,10^. As philosopher Patrick Lin said, “No one wants a car to care about greater good, they want a car to care about themselves”^11,12^. However, Deontology ignores consequence and utility, which makes decision-making difficult to be accepted by human. Therefore, there are drawbacks and advantages in each rule, so it is not good idea to adopt a unified rule in decision-making. Contissa et al. proposed a “moral knob” architecture for AV, where both ends of the knob correspond to “altruism” and “egoism” respectively, and the center corresponds to “complete neutrality”^13^. This customized decision-making rule is conducive to manage and deal with traffic accidents to avoid the occurrence of disputes over responsibility in traffic accidents^14^. However, this customized decision-making strategy may ignore others or collective interests for self-interest, which leads to prisoner’s dilemma. Rawls put forward Max–Min rule to conduct risk assessment for passengers and pedestrians in ethical dilemmas to make decision-making that can improve survival rate and maximize interests in ethical dilemmas^15–17^. However, it is hard for AV to accurately evaluate survival rate of all stakeholders in traffic accident. Moreover, the road user with higher survival rate is chosen as sacrificer to make people with better safe facilities be subject to greater risk. In addition, Dietmar and Lucie proposed social contract to solve moral dilemma, which is explicitly designed to derive standard acceptable to all rational agents and eliminates some intractable ethical disagreement^18^. However, it is hard to form a unified social contract around the world, which should be suitable for all people. In addition, a few scholars studied decision-making mechanism and methods in ethical dilemmas for AV. For example, Hong et al. proposed a decision-making platform (LO-MPC) to solve ethical dilemma. AV would make decision-making on the basis of priority and performance restriction, which aim to protect species with higher priority without violating performance restriction^19^. Wu and Lin proposed to integrate human morality with machine’s decision-making, which prevent machine from violating human morality and strategy by reinforcement learning^20^. Evans et al. put forward EVT (Ethical Valence Theory) in decision-making, which provided a mechanism that can keep trade-off among all road users^21^. AV makes decision by ethical valence and injury consequence, and ethical valence is measured on the basis of age, social status, gender et al. In general, pedestrian with higher ethical valence is given more priority to be protected when passengers in AV are not seriously injured. Geisslinger et al. proposed ethics of risk in decision-making, which includes three rules: Bayes rule, Max–Min rule and Equality rule. Because decision-making cannot meet multiple requirements by single rule, the three rules are integrated to make decision in moral dilemmas ^22,23^.

In all, a few factors can be explored by theoretical research and survey, which may have some influences on decision-making in ethical dilemma, such as number, rules et al. However, the weight and role of each factor cannot be determined and measured in decision-making, which is still not conducive for AV to make right decision-making in ethical dilemmas. Moreover, a single and few factors are considered in the above rules and architectures, so decision-making may be unreasonable and cannot be suitable for moral requirements. In the end, due to the lack of weight of each factor in decision-making, a few present decision-making mechanisms and models can only make qualitative decisions-making, but cannot make quantitative decision-making in ethical dilemmas, resulting in fuzziness and uncertainty of decision-making. Therefore, it is necessary and meaningful to study and evaluate the weight and role of each factor in decision-making to construct a computational decision-making models for AV.

## EWM theory

EWM is a mathematical method to synthesize all factors based on amount of information provided by various factors, and the weight of each factor in decision-making system is calculated on the basis of amount of information conveyed to decision-making system by each factor^24^. In general, the magnitude of feature variability can determine the weight of feature factors in the evaluation system. The smaller information entropy of a factor, the greater variation of the factor, the more information it provides, the greater the role it can play in comprehensive evaluation, the higher the weight in decision-making for AV. Therefore, the role and weight of each factor in decision-making can be calculated by EWM and AVWEWM. There are three steps to solve the weight of each factor in EWM.

### Data standardization

Supposing there are n samples in training set and k attributes in a sample, the jth attribute value of the ith sample is described by X_ij_ (i = 1,2,…,n; j = 1,2,…,k), then their standardized values ${Y}_{ij}$ may be calculated by Formula (1).

1 $${Y}_{ij}= \frac{{X}_{ij}-{\text{min}} ({X}_{i})}{{\text{max}} ({X}_{i}) - {\text{min}} ({X}_{i})}.$$

${Y}_{ij}$ indicates the standardized value of jth attribute on the ith sample. X_i_ indicates any sample in training set. ${\text{max}} ({X}_{i})$ and ${\text{min}} ({X}_{i})$ indicate the maximum and minimum value for an attribute in training set, so they indicate the maximum and minimum attribute value for the jth attribute in Formula (1).

### Solving information entropy

Firstly, the proportion ${{\text{p}}}_{{\text{ij}}}$ of standardized value in the ith sample to the sum of standardized values for all samples in training set is calculated for the jth attribute by Formula (2).

2 $${{\text{p}}}_{{\text{ij}}} = {{\text{Y}}}_{{\text{ij}}}\bigg/\sum_{{\text{i}}=1}^{{\text{n}}}{{\text{Y}}}_{{\text{ij}}}.$$

Then information entropy ${{\text{E}}}_{{\text{j}}}$ of the jth attribute can be calculated by Formula (3).

3 $${{\text{E}}}_{{\text{j}}}=-\frac{1}{lnn}\sum_{i=1}^{n}{p}_{ij}{\text{ln}}{p}_{ij},$$

when p_ij_ = 0, ${p}_{ij}\ln{p}_{ij} =0$ is defined in EWM.

### Solving weight

According to information entropy, the weight of each factor may be calculated  and measured in decision-making by Formula (4).

4 $${{\text{w}}}_{{\text{i}}}= \frac{1-{{\text{E}}}_{{\text{j}}}}{{\text{k}}-\sum {{\text{E}}}_{{\text{j}}}} \left(\mathrm{j }=\mathrm{1,2},...,{\text{k}}\right).$$

## Major factors extraction

According to present research literature and investigation on decision-making in ethical dilemma, there are many influencing factors in decision-making, including personal factors, organizational factors and objective factors. Personal factors include morality, age, gender, culture and fitness etc. Organizational factors refer to circumstance, system, law, punishment, reward, scale and market et al. Objective factors refer to vehicle performance, velocity, species, volume, lane width, harm and weather et al. At present, a few scholars explored personal factors in decision-making, which focus on age, career, gender, social status and fitness. However, taking personal factors into account may lead to discrimination in decision-making, which runs counter to fairness. Therefore, organizational and objective factors should be considered as much as possible in decision-making for AV.

**Figure 1:**
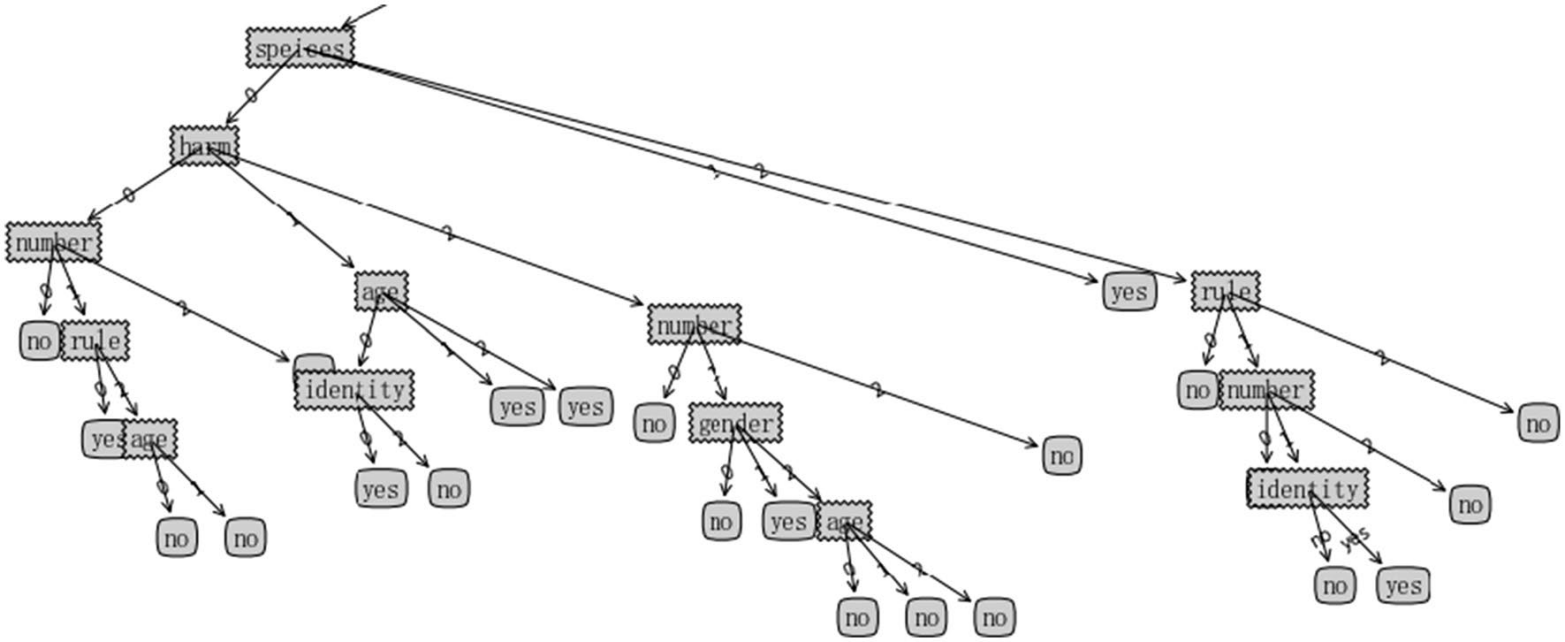
An ethical decision-making tree model based on ID3.0.

When too many factors are considered in decision-making for AV, information overlap and interference caused by multi-variable and multi-collinearity may occur in decision-making^25,26^, which causes complexity in decision-making model and architecture. Therefore, a few major factors are extracted and determined in decision-making by information and dependency measurement. The role and importance are reflected by information entropy. In general, the smaller information entropy of an attribute, the greater influence on decision-making. Therefore, a decision-making tree can be established by information entropy gain. Some attributes appearing in the common path of decision-making tree are chosen as major factors. In order to construct the decision-making tree model, 82 dilemmas are designed as training set, and 7 attributes including gender, age, number, rule, harm, social status and species are considered in the decision-making tree. For simplicity, going straight and swerving are set as two decision-making in ethical dilemmas. Moreover, each respondent randomly selects 15 moral dilemmas from 82 to be investigated by online, and 844 valid questionnaires are taken back and 12,660 data items are collected to calculate information entropy and information entropy gain of each attribute. Lastly, an ethical decision-making tree model is constructed based on information entropy gain by ID3.0 algorithm, just as shown in Fig. 1.

In order to completely show all attributes in decision-making tree model, social status is expressed by one word “identity”, traffic laws is expressed by one word “rule”. “Yes” shows AV chooses to swerve to protect road users on the default lane, “no” shows AV chooses to go straight to hit road users on the default lane.

It can be seen that species, traffic laws, number and harm are in the common path of the decision-making tree in Fig. 1, which indicates that 4 attributes have greater influences on decision-making in ethical dilemmas, and social status, age and gender have little influence.

Dependence measurement is also evaluated by mutual information between attribute and class variable. According to 1,048,575 survey data published in Moral Machine, the mutual information between each attribute and two classes and their differences may be calculated, which are shown in Table 1.

**Table 1:** Statistics table of mutual information between each attribute and two classes.

Feature	Gender		Number		Age		Social status		Traffic laws		Fitness	
Attribute	Female	Male	More	Less	Young	Old	High	Low	Yes	No	Good	Not
Swerving	0.509	0.365	0.751	0.197	0.736	0.202	0.683	0.294	0.748	0.199	0.534	0.357
Straight	0.491	0.635	0.249	0.803	0.264	0.798	0.317	0.706	0.252	0.801	0.466	0.643
Difference	0.018	− 0.27	0.502	− 0.606	0.472	− 0.596	0.366	− 0.412	0.496	− 0.602	0.068	− 0.286

In general, the more the difference of mutual information between attribute and class variable, the greater the influence on decision-making. For example, the mutual information between female and swerving is 0.509 and that between female and straight is 0.491, it indicates that 50.9% respondents wish AV swerves and 49.1% wish AV goes straight in the moral dilemma. The difference between both mutual information is only 0.018 for female, which indicates that gender has little influence on decision-making in ethical dilemma. However, for number, the mutual information between more attribute and swerving is 0.751 and that between more attribute and straight is 0.249, their difference is 0.502, which shows that number has great influences on decision-making in ethical dilemma. According to statistics in Table 1, it indicates that number, age, traffic laws have more influences on decision-making. On the contrary, social status, fitness and gender have less influence. For species, the mutual information between animal and class variables is not calculated by survey data in Moral Machine, so species is not listed in Table 1. However, most respondents wish that human should be given to more priority to be protected on the basis of survey data in Moral Machine. In addition, fitness indicates people’s characters are more fit, for example, Male/Female Athlete and normal Man/Woman. Or else, another is less fit, for example, non-athletes and large Man/Woman.

According to the above analysis and measurement, species, harm, traffic laws, number and age, are extracted as major factors in decision-making in ethical dilemmas at last. Although age, as personal factor, causes some discrimination against the elderly and disputes in decision-making. However, most respondents still like to give more priority to protect young in an inevitable accident, even many old peoples wish to protect the young in ethical dilemmas. Therefore, age is also regarded as a major factor in decision-making.

## AVWEWM decision-making model

In order to solve and evaluate the weight and role of each major factor in AVWEWM and EWM model, 116 moral dilemmas are designed to be surveyed for teachers, students and their families over 18 years old, where there are one or more major attributes as far as possible. A total of 1006 valid questionnaires are received to collect 15,090 data items. The decision-making in all dilemmas are determined by majority principle. Furthermore, 84 are chosen as training set to construct decision-making model from 116 moral dilemmas. Secondly, attribute values and attribute vector are quantified by a uniform quantization rule. Thirdly, according to the proportion of choosing to go straight or swerve in a dilemma, attribute values for all attributes are weighted by the proportion to get an actual attribute value and attribute vector R, then each actual attribute value is standardized to establish standardization set Y by data standardization k, Fourthly, information entropy of each attribute is calculated by the proportion of standardized value in a dilemma to the sum of standardized values for all dilemmas in training set by function f. Lastly, the weight w_i_ in decision-making is solved for each attribute, then AVWEWM model F is constructed to deal with ethical dilemmas for AV by function φ.

$${\text{T}}\stackrel{{\text{s}}}{\to }\mathrm{Q }\stackrel{w}{\to }\mathrm{ R }\stackrel{k}{\to }\mathrm{ Y }\stackrel{f}{\to }\mathrm{ E }\stackrel{\varphi }{\to }\mathrm{ F}.$$

### Quantification ($\mathbf{T}\stackrel{\mathbf{s}}{\to }$ Q)

It is hard task for AV to make precise quantitation for an attribute because of detection technology. For example, it is hard and unnecessary to accurately identify the age of pedestrians and passengers by detection module in AV. Therefore, an attribute is quantified by comparing the attributes between two parties in ethical dilemma to get attribute value. In general, when a party has an attribute which is conducive to be protector, the attribute value is quantified as 2. On the contrary, when a party has an attribute that is not conducive to be protect, the attribute value is quantified as 0. Otherwise, when an attribute is the same or similar between two parties, the attribute value is quantified as 1, just as shown in Formula (5).

5 $${\text{Q}}_{i} = \left\{ {\begin{array}{*{20}l} 0 \hfill & {\text{Attribute is not conducive to be protector for a party}} \hfill \\ 1 \hfill & {\text{The same or similar attribute in both parties}} \hfill \\ 2 \hfill & {\text{Attribute is conducive to be protector for a party}} \hfill \\ \end{array} } \right..$$

**Figure 2:**
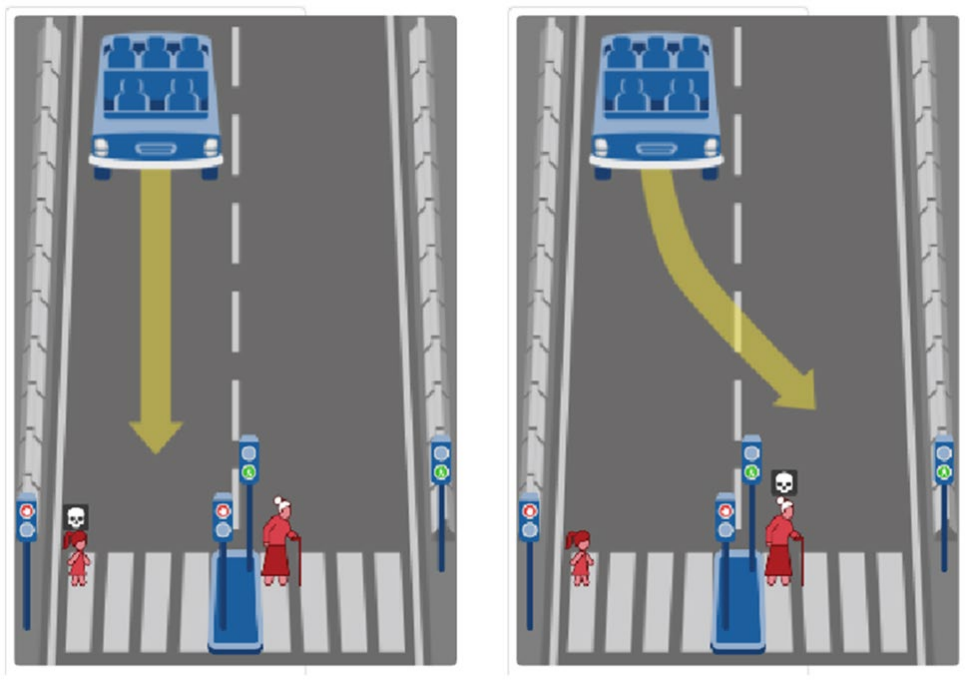
A ethical dilemma.

For example, there is an ethical dilemma in Fig. 2, a little girl is crossing the road at a red light in the straight lane and an old lady is crossing the road at a green light in the swerving lane. According to survey, 86.36% respondents choose to go straight and only 13.64% choose to swerve in the dilemma, which indicates that the old lady in the swerving lane is chose as protector. There are two different attributes including age and traffic laws between the little girl in the straight and the old lady in the swerving. According to survey results, the older the age, the more not conducive to be protected. Therefore, the attribute value is 0 in the swerving lane for age. For traffic laws, the more compliance with traffic laws, the more conducive to be protected, the attribute value is 2 in the swerving lane. Furthermore, other major attributes such as number, harm and species are the same between straight and swerving, their attribute values are all 1. Therefore, The attribute set in the straight lane Q_s_ = {1, 1,0,1,2}, on the contrary, the attribute set in the swerving lane Qt = {1,1,2,1,0}.

### Weighting attribute value (Q $\stackrel{\mathbf{w}}{\to }$ R)

Since decision-making result is determined by majority principle in ethical dilemma, the weight in decision-making model should be calculated because original attribute value for each attribute may not reflect accurately human decision-making result. Therefore, the attribute value should be weighted by the proportion p_i_ of choosing to swerve or go straight to get an actual attribute value and attribute set R as shown in Formula (6).

6 $${{\text{R}}}_{{\text{i}}} = {{\text{p}}}_{{\text{i}}}{{\text{Q}}}_{{\text{i}}}.$$

For the ethical dilemma in Fig. 2, since the proportion of choosing to go straight is 86.36%, the actual attribute set R for the swerving lane in the dilemma is {0.8636, 0.8636, 1.7272, 0.8636, 0}. The same way is also used to calculate actual attribute sets in other training dilemmas.

### Data standardization (R $\stackrel{{\varvec{k}}}{\to }$ Y)

Since each actual attribute value is different in each dilemma, it cannot reflect the role and importance of each attribute in decision-making. Therefore, these original attribute values need to be processed by data standardization by Formula (1). Firstly, maximum and minimum actual attribute values are sought for each attribute in all training samples. According to statistical results, maximum and minimum actual attribute values for 5 attributes are shown in Table 2. Since human is give more priority to be protected in these moral dilemmas, the actual minimum attribute value is 0.511 for species.

**Table 2:** Statistical table of maximum and minimum actual attribute value for each attribute.

Factor	Species	Harm	Traffic laws	Number	Age
Max (R_i_)	1.954	1.932	1.954	1.932	1.91
Min (R_i_)	0.511	0	0	0	0

The standardization of each actual attribute value is calculated by Formula (1) to generate standardization data set Y.

### Information entropy (Y $\stackrel{{\varvec{f}}}{\to }$ E)

Before solving information entropy of each attribute, the proportion of standardized value in a moral dilemma to the sum of standardized values in all moral dilemmas should be calculated for each attribute by Formula (2). Then information Entropy E_j_ of each attribute is solved by Formula (3), which is shown in Table 3.

**Table 3:** Statistical table of information entropy for each attribute.

Factor	Species	Harm	Traffic laws	Number	Age
EWM (E_j_)	0.8488	0.9004	0.9197	0.9074	0.9513
AVWEWM (E_j_)	0.8175	0.8251	0.8487	0.938	0.96

As can be seen in Table 3, there are a few differences in information entropy calculated by EWM and AVWEWM for 5 major attributes. Information entropy of traffic laws in AVWEWM is more than that of number in EWM. However, information entropy of traffic laws is less than that of number in AVWEWM.

### Weight solving and model construction (E $\stackrel{\boldsymbol{\varphi }}{\to }$ F)

According to information entropy in Table 3, the weight w_i_ of each attribute may be solved in decision-making by formula (4). Furthermore, both weights of each attribute are different in EWM and AVWEWM, just as shown in Table 4.

**Table 4:** Weight statistical table for each attribute.

Attribute	Species	Harm	Traffic laws	Number	Age
EWM (w_i_)	0.3201	0.2108	0.17	0.196	0.1031
AVWEWM (w_i_)	0.2988	0.2864	0.2477	0.1015	0.0655

**Figure 3:**
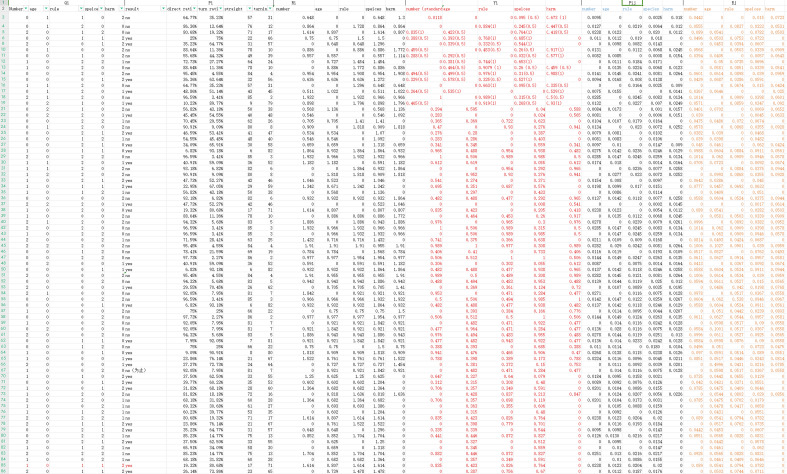
Screenshots of data processing form for training cases.

In order to clarify the method of processing data in these dilemma cases, a screenshots of detailed data processing forms for these training cases is shown as Fig. 3.

There are six values including quantification, survey result, weighting attribute value, data standardization, information entropy for each attribute in this form. In the end, weight for 5 major factors can be calculated.

Lastly two decision-making models for moral dilemma are constructed by EWM and AVWEWM for AV respectively, by which the risk degrees F of going straight and swerving are calculated by Formula (7).

7 $$\mathrm{F }= \sum \limits_{i=1}^{k}{w}_{i}{Q}_{i}.$$

When the risk degree of going straight (Fs) is more than the risk degree of swerving (Ft), AV should choose to swerve for protecting road users in the straight. For example, the attribute vector for the straight and the swerving are {1,1,0,1,2} and {1,1,2,1,0} respectively in the dilemma in Fig. 2. According to Formula (7), the Fs and Ft of going straight and swerving may be calculated in EWM and AVWEWM.

EWM:

$${\text{going straight}}:{\text{ Fs }} = \, 0.{32}0{1} \times {1 } + \, 0.{21}0{8} \times {1 } + \, 0.{17} \times 0 \, + \, 0.{196} \times {1 } + \, 0.{1}0{31} \times {2 } = \, 0.{9331},$$

$${\text{swerving}}:{\text{ Ft }} = \, 0.{32}0{1} \times {1 } + \, 0.{21}0{8} \times {1} + \, 0.{17} \times {2 } + \, 0.{196} \times {1 } + \, 0.{1}0{31} \times 0 \, = { 1}.0{669}.$$

Because of Ft > Fs, AV should choose to go straight to hit the little girl in the straight lane, which is consistent with public opinion.

AVWEWM:

$${\text{going straight}}:{\text{ Fs }} = \, 0.{2988} \times {1 } + \, 0.{2864} \times {1 } + \, 0.{2477} \times 0 \, + 0.{1}0{15} \times {1 } + \, 0.0{655} \times {2 } = \, 0.{8177},$$

$${\text{swerving}}:{\text{ Ft }} = \, 0.{2988} \times {1 } + \, 0.{2864} \times {1 } + \, 0.{2477} \times {2 } + \, 0.{1}0{15} \times {1 } + \, 0.0{655} \times 0 \, = { 1}.{1821}.$$

Because of Ft > Fs, AV should choose the same decision-making as EWM.

## Test and discussion

In order to compare both models based on EWM and AVWEWM, 40 ethical dilemmas are chosen to test EWM and AVWEWM respectively from 116, test results are shown in Table 5.

**Table 5:** Test results statistical table.

No.	1	2	3	4	5	6	7	8	9	10	11	12
EWM	0.015	− 0.134	− 0.692	0.559	− 0.173	− 0.215	− 0.082	− 0.167	0.052	0.219	0.258	0.288
AVWEWM	0.041	− 0.364	− 0.305	0.520	− 0.178	− 0.442	− 0.077	− 0.317	− 0.292	0.025	− 0.161	0.208
Public	Y	N	N	Y	Y	N	N	N	N	Y	N	Y

No	13	14	15	16	17	18	19	20	21	22	23	24
EWM	− 0.526	0.555	− 0.288	− 0.125	− 0.559	− 0.555	− 0.121	0.121	0.300	0.680	− 0.163	− 0.576
AVWEWM	− 0.567	0.937	− 0.208	− 0.054	− 0.520	− 0.937	− 0.471	0.471	0.102	0.411	− 0.734	− 0.996
Public	N	Y	N	Y	N	N	Y	Y	Y	Y	N	N

No.	25	26	27	28	29	30	31	32	33	34	35	36
EWM	0.271	0.951	0.513	− 0.029	− 0.947	− 0.588	− 0.611	− 0.300	0.215	0.029	− 0.186	− 0.029
AVWEWM	− 0.268	0.723	0.674	− 0.370	− 1.140	− 0.890	− 0.228	− 0.102	0.442	0.370	− 0.072	− 0.37
Public	Y	Y	Y	N	N	N	N	Y	Y	N	N	N

No	37	38	39	40								
EWM	− 0.248	− 0.300	0.029	− 0.248								
AVWEWM	− 0.395	− 0.102	0.370	− 0.395								
Public	N	N	Y	N								

In the above table, the values in EWM and AVWEWM describe the differences between Fs and Ft for EWM and AVWEWM respectively for a dilemma case. If the value is positive, AV chooses to swerve in the dilemma. Or else, AV chooses to go straight. Moreover, the value in Public describes public opinion. Y indicates that most respondents choose to swerve in the test dilemma, and N indicates that most respondents choose to go straight in the test dilemma. According to test results, the decision-making in 34 test dilemmas are consistent with public opinions by EWM, its accuracy is 85%. However, the decision-making in 35 moral dilemmas are consistent with public opinions by AVWEWM, its accuracy is 87.5%. Therefore, it indicates that AVWEWM is more suitable for public requirement than EWM. Furthermore, the decision-making of test case 5, 16, 19 and 32 by EWM and AVWEWM are both different from public opinions, and there are differences in species and harm for these test cases. When another attribute, such as number or traffic laws, is also different between straight and swerving, it is possible to make different decision-making from public opinion by EWM and AVWEWM. In addition, the decision-making in case 9 and 11 by AVWEWM are consistent with public opinions and those by EWM are not, it demonstrates that weighted attribute value can better suitable for human requirement. Most respondents wish that these pedestrians obeying traffic laws should be given more priority to be protected in ethical dilemmas. Because the weight of species in AVWEWM is less than that in EWM, and the weight of harm in AVWEWM is more than that in EWM, the decision-making in case 25 by AVWEWM is different from that by EWM and public opinion, because human is not given more priority to be protected in AVWEWM than EWM.

The weight of species is the largest in both EWM and AVWEWM, which indicates that species has great role and importance on decision-making in moral dilemmas. Furthermore, priority can be given to protect human in most dilemmas, the weight of species may be underestimated in this paper. The largest weight indicates that both EWM and AVWEWM comply with Asimov’s Three Laws of Robotics and moral norms, because human has dual attributes, including natural attribute and social attribute, and have the capacity of understanding and changing the world. The fact that animals or inanimate cannot means that human have higher social value than animals or inanimate and should be protected preferentially. The weight of harm is the second in decision-making, but the weight in AVWEWM is higher than that in EWM, which indicates that the former pays more attention to injury consequence in decision-making. It can try to reduce harm caused by traffic accidents and promote science and technology to be good. However, the weight of traffic laws is different in EWM and AVWEWM, and the weight in EWM is relatively small, even less than that of number, which shows that AVWEWM pays more attention to complying with traffic laws for respondents. It may be more reasonable in real life, for only strengthening comply with traffic laws, can prevent AV using fully their own decision-making mechanisms and rules replace of legal justice, keep a balance between self decision-making and law enforcement^27^. It may implement decision-making under a legal framework to ensure fairness and rationality in ethical dilemmas for AV. Furthermore, the weight of number in EWM is higher than that in AVWEWM, which can prevent more pedestrians from being injured in traffic accident. However, it may be unreasonable to overstate the weight of number in decision-making, which can violate the rights of fewer people for protecting more. Therefore, it may be more reasonable to pay more attention to number in a serious accident. For age, since it is a personal factor, high weight should not be set in decision-making. However, the weight of age in EWM is more 10%, which is unreasonable.

## Conclusions

According to survey data, EWM and AVWEWM are used to solve the weight of 5 major factors to evaluate their roles and importance in decision-making, which can provide a condition for establishing a computational decision-making model in the future. Moreover, the weight and role of each factor can provide a few references for traffic management and legal departments to formulate laws and regulations relative to AV. However, due to the simplification in quantization, the accuracy and liability of weight is affected. In addition, only 5 major factors are extracted and considered in this paper, a few other important factors may be ignored, so it is necessary to explore other factors in the future.

### Ethical approval

All methods were carried out in accordance with relevant guidelines and regulations. All informed consent was obtained from all subjects. All subjects were informed in advance about the purpose, tasks. All experimental protocols were approved by China Association for Ethical Studies (CAES).

## Data Availability

The data used to support the findings of this study are available from the corresponding author upon request.
